# SELECTpro: effective protein model selection using a structure-based energy function resistant to BLUNDERs

**DOI:** 10.1186/1472-6807-8-52

**Published:** 2008-12-03

**Authors:** Arlo Randall, Pierre Baldi

**Affiliations:** 1 School of Information and Computer Sciences, University of California, Irvine, CA, 92697, USA; 2 Institute for Genomics and Bioinformatics, University of California, Irvine, CA, 92697, USA

## Abstract

**Background:**

Protein tertiary structure prediction is a fundamental problem in computational biology and identifying the most native-like model from a set of predicted models is a key sub-problem. Consensus methods work well when the redundant models in the set are the most native-like, but fail when the most native-like model is unique. In contrast, structure-based methods score models independently and can be applied to model sets of any size and redundancy level. Additionally, structure-based methods have a variety of important applications including analogous fold recognition, refinement of sequence-structure alignments, and de novo prediction. The purpose of this work was to develop a structure-based model selection method based on predicted structural features that could be applied successfully to any set of models.

**Results:**

Here we introduce SELECTpro, a novel structure-based model selection method derived from an energy function comprising physical, statistical, and predicted structural terms. Novel and unique energy terms include predicted secondary structure, predicted solvent accessibility, predicted contact map, β-strand pairing, and side-chain hydrogen bonding.

SELECTpro participated in the new model quality assessment (QA) category in CASP7, submitting predictions for all 95 targets and achieved top results. The average difference in GDT-TS between models ranked first by SELECTpro and the most native-like model was 5.07. This GDT-TS difference was less than 1% of the GDT-TS of the most native-like model for 18 targets, and less than 10% for 66 targets. SELECTpro also ranked the single most native-like first for 15 targets, in the top five for 39 targets, and in the top ten for 53 targets, more often than any other method. Because the ranking metric is skewed by model redundancy and ignores poor models with a better ranking than the most native-like model, the BLUNDER metric is introduced to overcome these limitations. SELECTpro is also evaluated on a recent benchmark set of 16 small proteins with large decoy sets of 12500 to 20000 models for each protein, where it outperforms the benchmarked method (I-TASSER).

**Conclusion:**

SELECTpro is an effective model selection method that scores models independently and is appropriate for use on any model set. SELECTpro is available for download as a stand alone application at: . SELECTpro is also available as a public server at the same site.

## Background

Selecting the most native-like model from a set of possible models is a crucial task in protein structure prediction. A variety of Model Quality Assessment Programs (MQAPs) have been developed that assign numeric scores to models in a set, and then use the scores to rank the models and ultimately select a single model. MQAP methods can be divided roughly into three categories based on the type of information they use: evolutionary methods use sequence or profile similarity between target sequence and template, consensus methods use similarity between models, and structure-based methods use model coordinates [1]. Each category of methods has inherent strengths and weaknesses.

Evolutionary methods can provide quality scores that have been shown to correlate with structural similarity to native [2]. However, for lower confidence alignments the scores do not correlate well with structural similarity. Furthermore, identification of the best template and specific alignment can be difficult. In addition, models built from multiple templates or template-free methods cannot be scored appropriately by evolutionary methods alone.

Consensus methods take advantage of the observation that similar models produced by different predictors tend to be more accurate than those that are structural outliers. In practice, consensus methods outperform the methods they draw from, and they rarely pick a very poor model. The disadvantage, however, is that when the best model is a structural outlier it will be overlooked for lack of popularity [1]. Also, consensus methods are not appropriate for selecting from small sets of structurally diverse models, especially in the extreme case of a two-model set.

While consensus methods depend on similarity between models, structure-based methods calculate scores on each model independently. For this reason, structure-based methods can be applied to model sets of any size and diversity, and will produce the same score for a model regardless of the other models in the set. Structure-based methods can also be used for template-free modeling [3-6] and model refinement procedures [7,8]. One weakness of high resolution structure-based methods, including protein free energy approximation functions [9-12] and physics based approaches [13,14], is their sensitivity to local structural irregularities such as steric clashes and chain breaks, which can significantly bias scores on otherwise accurate models. Even slight differences in model backbones can produce significantly different scores [15]. Lower resolution structure-based methods, such as statistical potentials [6,16,17], are more robust to backbone variation, but are sensitive to extended low contact-order regions in the models.

Here we describe SELECTpro, a novel structure-based MQAP that combines high and low resolution energy terms into a model selection method that is effective on model sets of variable size, diversity, and target difficulty. Most of our assessment is calculated from the CASP7 model quality assessment category (QA) results published online [18]. The QA category provides a framework for the unbiased evaluation of MQAPs on ensembles of models produced by diverse automated prediction methods.

## Results and discussion

We analyze the CASP7 quality assessment category predictions with a focus on the quality of the model ranked first by each predictor and the recovery of the most native-like model in the set. Only *SetAll *is used in the assessment of the quality of the model ranked first by each group (Table 1). The results are very similar when using *SetComplete *(data not shown) because QA groups rarely rank an incomplete model first.

**Table 1:** Quality of Model Ranked First (M_QA1_) Relative to Most Native-Like Model (M_max_)

		**Summary Results**				**Common Subset Results**				
**Group**	**Targets **^a^	Δ*GDT*_*QA*1 _= 0	Δ*GDT*_*QA*1% _< 1	Δ*GDT*_*QA*1% _< 10	$\bar{\Delta GDT_{QA1}}$	Δ*GDT*_*QA*1 _= 0	Δ*GDT*_*QA*1% _< 1	Δ*GDT*_*QA*1% _< 10	$\bar{\Delta GDT_{QA1}}$	*p-value*
**699_1**	**95 (124)**	**12**	**18**	**66**	**5.07**	**-**	**-**	**-**	**-**	**-**
713_1	95 (124)	7	11	63	5.44	**12**	**18**	**66**	**5.07**	2.5E-01
634_1	95 (124)	7	15	53	7.75	**12**	**18**	**66**	**5.07**	**1.6E-03**
704_1	95 (124)	5	8	49	7.76	**12**	**18**	**66**	**5.07**	**3.5E-04**
178_1	95 (124)	8	12	59	8.44	**12**	**18**	**66**	**5.07**	**3.0E-03**
633_1	95 (124)	6	9	52	10.12	**12**	**18**	**66**	**5.07**	**1.8E-06**
692_1	95 (124)	6	9	52	10.16	**12**	**18**	**66**	**5.07**	**1.2E-06**
657_1	95 (124)	1	5	40	12.71	**12**	**18**	**66**	**5.07**	**1.8E-08**
691_1	95 (124)	0	1	24	15.10	**12**	**18**	**66**	**5.07**	**2.2E-13**
091_1	94 (123)	11	18	61	7.93	**12**	**18**	**65**	**5.10**	**2.1E-03**
026_1	94 (123)	1	2	40	9.30	**12**	**18**	**65**	**5.10**	**1.2E-07**
338_5	93 (122)	2	3	37	15.10	**12**	**18**	**65**	**5.05**	**1.3E-09**
556_1	93 (121)	10	15	51	6.83	**12**	**18**	**64**	**5.15**	**1.8E-02**
734_1	92 (120)	4	4	36	16.16	**12**	**18**	**64**	**5.10**	**5.6E-11**
718_1	92 (119)	1	3	32	14.04	**11**	**17**	**64**	**5.19**	**1.6E-10**
717_1	87 (112)	3	7	36	10.15	**10**	**15**	**59**	**5.31**	**4.3E-08**
016_1	86 (111)	5	9	49	7.93	**10**	**16**	**58**	**5.26**	**1.4E-03**
038_1	85 (108)	3	7	60	5.75	**11**	**16**	**58**	**5.34**	1.2E-01
276_1	80 (104)	5	5	39	8.94	**11**	**17**	**54**	**5.21**	**7.7E-07**
013_1	78 (100)	4	6	41	9.86	**10**	**15**	**56**	**4.87**	**2.0E-05**
703_1	69 (86)	3	6	35	8.74	**9**	**15**	**45**	**5.35**	**1.2E-04**
191_1	61 (78)	2	5	32	9.35	**7**	**10**	**39**	**6.04**	**2.3E-03**
066_1	55 (72)	1	2	14	23.19	**7**	**10**	**45**	**4.09**	**4.3E-10**

^a ^The number of targets where the QA group made a valid prediction (*N*_*T*_) with the number of domains of these targets (*N*_*D*_) in parentheses.

* SELECTpro (699_1) results appear in bold face and all results that are better than SELECTpro are underlined. Statistically significant p-values (p < .05) are also in bold.

**Figure 1:**
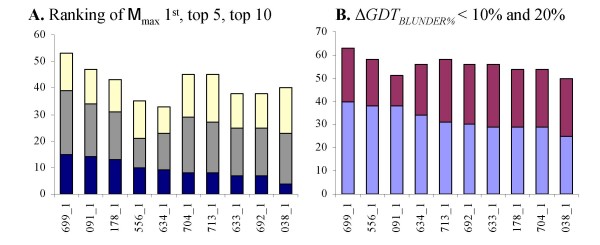
**Recovery of M_max _using *SetComplete***. (**A**) number of targets where M_max _is ranked first (top of dark blue bar), in the top five (top of gray bar), and in the top ten (top of white bar). (**B**) number of targets where Δ*GDT*_*BLUNDER*% _is less than 10% (top of light blue bar) and less than 20% (top of purple bar). Only the first ten groups are shown in both graphs.

The assessment of the recovery of the most native-like model, is performed on both *SetAll *and *SetComplete *(Table 2) because the few cases where an incomplete model is the most native-like have a significant effect on the average recovery metrics of all QA groups. Incomplete and irregular models are especially challenging for structure-based methods. A comparison of the average Pearson Correlation on *SetAll *and *SetComplete*, highlights these issues (Table 3). The frequency of recovering the most native-like model is calculated on *SetComplete *(Figure 1).

**Table 2:** Recovery of Top GDT-TS Model (M_max_)

		***SetAll***							***SetComplete***				
		**Summary Results**		**Common Subset Results**					**Summary Results s**		**Common Subset Results**		
**Group**	**Targets**^a^	$\bar{rank}$	$\bar{\Delta GDT_{BLUNDER}}$	$\bar{rank}$	$\bar{\Delta GDT_{BLUNDER}}$	*p-value*	**Group**	**Targets**	$\bar{rank}$	$\bar{\Delta GDT_{BLUNDER}}$	$\bar{rank}$	$\bar{\Delta GDT_{BLUNDER}}$	*p-value*

**699_1**^b^	**95 (124)**	**29.8**	**11.8**	**-**	**-**	-	**699_1**	**95 (124)**	**17.8**	**10.4**	-	-	-
704_1	95 (124)	46.5	17.8	**29.8**	**11.8**	**2.7E-06**	633_1	95 (124)	20.7	11.8	**17.8**	**10.4**	**4.7E-02**
178_1	95 (124)	42.3	19.6	**29.8**	**11.8**	**2.9E-04**	634_1	95 (124)	29.5	12.7	**17.8**	**10.4**	5.7E-02
657_1	95 (124)	78.5	37.0	**29.8**	**11.8**	**3.9E-20**	704_1	95 (124)	24.1	13.1	**17.8**	**10.4**	**1.1E-02**
634_1	94 (121)	52.0	16.5	**29.2**	**11.7**	**1.3E-02**	178_1	95 (124)	24.1	13.7	**17.8**	**10.4**	**6.5E-03**
091_1	94 (123)	27.2	17.4	**29.3**	**11.9**	**2.2E-05**	657_1	95 (124)	53.5	32.0	**17.8**	**10.4**	**8.6E-18**
633_1	94 (121)	39.0	20.6	**29.2**	**11.7**	**1.3E-08**	713_1	94 (122)	18.3	10.9	**17.9**	**10.4**	2.0E-01
026_1	94 (123)	55.9	22.7	**29.4**	**11.6**	**3.2E-10**	692_1	94 (122)	20.6	11.6	**17.7**	**10.3**	6.7E-02
556_1	93 (121)	33.8	11.7	**29.0**	**11.7**	*	091_1	94 (123)	16.8	12.3	**17.4**	**10.4**	**2.4E-02**
692_1	93 (119)	38.7	20.6	**29.2**	**11.6**	**1.1E-08**	026_1	94 (123)	37.3	18.3	**17.6**	**10.2**	**1.5E-07**
691_1	93 (120)	98.1	28.6	**28.6**	**11.7**	**9.6E-19**	691_1	94 (123)	54.4	22.2	**17.4**	**10.4**	**2.4E-14**
338_2	93 (122)	60.4	30.2	**30.2**	**11.9**	**2.7E-15**	556_1	93 (121)	21.2	10.3	**17.2**	**10.2**	4.9E-01
713_1	92 (116)	26.4	12.8	**29.6**	**11.8**	3.2E-01	338_2	93 (122)	28.2	16.8	**18.0**	**10.4**	**1.5E-08**
734_1	89 (116)	55.2	31.5	**29.3**	**11.2**	**1.6E-15**	734_1	88 (115)	28.9	18.1	**17.3**	**9.6**	**7.0E-09**
718_1	83 (105)	81.6	31.9	**30.5**	**12.0**	**1.6E-14**	718_1	83 (105)	46.4	26.9	**17.6**	**10.4**	**4.5E-13**
717_1	78 (98)	46.8	22.8	**30.9**	**12.0**	**3.4E-09**	717_1	78 (98)	28.4	16.4	**17.6**	**10.3**	**2.1E-05**
013_1	78 (100)	60.1	27.5	**30.2**	**12.0**	**1.5E-09**	013_1	78 (100)	32.4	17.6	**18.5**	**10.3**	**3.7E-06**
276_1	78 (102)	52.9	28.9	**29.6**	**11.6**	**3.3E-12**	276_1	78 (102)	29.0	18.7	**17.5**	**10.2**	**8.5E-10**
038_1	70 (87)	25.9	11.9	**27.6**	**11.8**	4.6E-01	038_1	74 (95)	19.8	10.7	**17.4**	**10.4**	3.6E-01
703_1	69 (86)	37.2	20.6	**31.5**	**11.9**	**5.1E-07**	703_1	69 (86)	20.6	14.5	**17.6**	**10.5**	**4.7E-04**
191_1	61 (78)	45.5	21.9	**26.2**	**12.6**	**1.0E-06**	191_1	61 (78)	27.6	15.2	**16.7**	**11.1**	**2.8E-03**
066_1	55 (72)	91.1	54.6	**30.5**	**10.5**	**5.1E-24**	066_1	55 (72)	48.0	46.5	**18.5**	**9.1**	**1.8E-18**
016_1	53 (72)	30.9	20.0	**31.2**	**12.5**	**2.0E-05**	016_1	53 (70)	18.2	18.5	**17.8**	**11.0**	**1.4E-05**

^a ^The number of targets where the QA predictor scored M_max _(*N*_*T*_) with the number of domains of these targets (*N*_*D*_) in parentheses.

^b ^In the CASP7 submission SELECTpro did not have a score for M_max _of target T0356 due to a processing error. We added in the score for this analysis in order to make complete common subset comparisons.

* SELECTpro (699_1) results appear in bold face and all results that are better than SELECTpro are underlined. Statistically significant p-values (p < .05) are also in bold.

**Table 3:** Correlation of Selected Groups

**Group**	**Targets**	***SetAll ***$\bar{PC}$	***SetComplete ***$\bar{PC}$	Δ*PC*
634_1 (Pcons) ^a^	95	0.811	0.847	0.036
713_1 (Circle-QA) ^b^	95	0.765	0.823	0.058
633_1 (ProQ) ^b^	95	0.716	0.781	0.064
699_1 (SELECTpro) ^b^	95	0.676	0.763	0.087
556_1 (LEE) ^c^	93	0.814	0.792	-0.023

^a ^Consensus method.

^b ^Structure based method.

^c ^QA scores calculated as GDT-TS similarity to human predictor of LEE.

**Figure 2:**
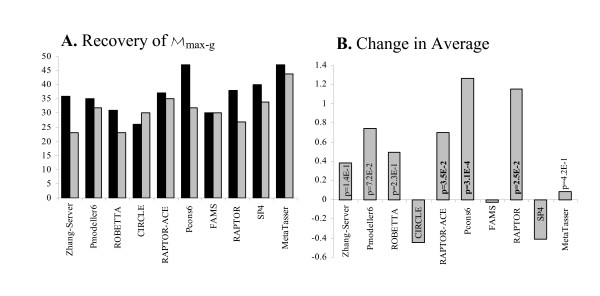
**Reranking models from top servers**. Each server predictor submitted five models per target, with the highest confidence model ranked first. (**A**) the number of targets where each server's highest GDT-TS model is ranked first is shown with gray bars, and black bars when the models are reranked with SELECTpro. (**B**) shows the change in average GDT-TS for each group when SELECTpro is used to select model 1. P-values of paired t-tests are shown above the horizontal axis when SELECTpro demonstrates improved model selection and statistically significant improvements (p < .05) are in bold.

**Figure 3:**
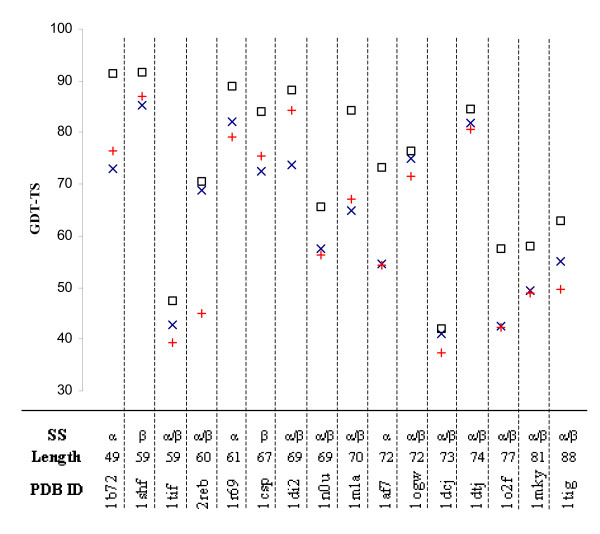
**Large Decoy Set Model Selection**. Large decoy set model selection with SELECTpro on I-TASSER benchmark set. This set of 16 small proteins was used as one of the benchmark sets for evaluating the I-TASSER method [19]. The complete decoy sets can be downloaded from [20]. Each protein has from 12500 to 20000 decoy models. For each protein different symbols are used to indicate the GDT-TS of M_max _(□), SELECTpro's M_*QA*1 _(×), and I-TASSER's M_*QA*1 _(+).

The utility of SELECTpro for selecting the best model from a small set is demonstrated by selecting from the five models submitted for each target by the top automated predictors. These small set selection results are calculated using *SetAll *(Figure 2). SELECTpro is also evaluated on a recent benchmark set of 16 small proteins with large decoy sets of 12500 to 20000 models for each protein and compared to I-TASSER (Figure 3).

To make fair comparisons to groups participating on only a subset of targets, common subset comparisons between SELECTpro and each of these groups are included in Tables 1 and 2. Only groups participating on at least half of the targets are included, and for groups with multiple submissions only the best one is shown. In the results tables any value that is better than SELECTpro is underlined.

For multiple domain targets, the sum of GDT-TS over all domains is used as the GDT-TS of the model. Since the QA predictions correspond to the entire structures, it is impossible to fairly assess the domains independently.

To assess the significance of the summary statistics compared in Table 1, Table 2, and Figure 2, we performed paired t-tests between SELECTpro each other group on common subsets of targets (or targets and models when appropriate). All p-values from the tests appear in the tables and figure, but only statistically significant p-values (p < .05) are shown in bold.

The following notations are used throughout the results section:

• M_max_: The model with the highest GDT-TS among all server models.

• M_*QA*1_: The model with the highest QA score.

• *N*_*T*_: The number of targets a group made valid predictions on.

• *N*_*D*_: The number of domains a group made valid predictions on.

The recovery of M_max _by a QA predictor can only be evaluated if M_max_was scored by the predictor. In most cases QA predictors did not provide scores for all available server models, and frequently there is no score for M_max_. For example, predictor 016_1 (AMBER/PB) made submissions on 86 targets, but M_max _is only scored for 53 of these targets – so only these targets (*N*_*T *_= 53) can be evaluated for this predictor.

### Quality of Model Ranked First (M_QA1_) Relative to Most Native-Like Model (M_max_)

In this section on the assessment of the model ranked first, and the corresponding Table 1, we use the following three metrics:

• Δ*GDT*_*QA1 *_= GDT-TS(M_max_) - GDT-TS(M_*QA*1_) : The GDT-TS difference between M_max _and M_*QA*1 _measures how much is lost by selecting M_*QA*1 _rather than M_max _for a single target.

• $\bar{\Delta GDT_{QA1}}$ = ΣΔ*GDT*_*QA*1_/*N*_*D *_: The average Δ*GDT*_*QA*1 _is a simple way of assessing the quality of M_*QA*1 _over all targets.

• Δ*GDT*_*QA*1% _= Δ*GDT*_*QA*1_/GDT-TS(M_max_) : The GDT-TS difference percentage allows for comparison across targets with different numbers of domains and difficulty levels.

The columns of Table 1 are: (1) group number; (2) number of targets the group made predictions on; (3) number of targets such that Δ*GDT*_*QA*1 _= 0; (4) number of targets such that Δ*GDT*_*QA*1% _< 1%; (5) number of targets such that Δ*GDT*_*QA*1% _< 10%; and (6) $\bar{\Delta GDT_{QA1}}$. The common subset results section has an additional column for the p-value of the paired t-test using Δ*GDT*_*QA*1_. The rows are sorted first by the number of targets and then by $\bar{\Delta GDT_{QA1}}$. Of the groups participating on all 95 targets, SELECTpro has the lowest average Δ*GDT*_*QA*1_, with a value of 5.07, followed closely by group 713_1 (Circle-QA), with a value of 5.44. Predictor 038_1 (GeneSilico) has an average Δ*GDT*_*QA*1 _of 5.75, with predictions on 85 targets. In common subset comparisons with these two groups SELECTpro is not significantly better, with p-values of .25 and .12 respectively. In common subset comparisons with all remaining groups SELECTpro is significantly better.

Another way to assess the quality of M_*QA*1 _over many targets is to count the number of targets such that M_*QA*1 _is the best model, or nearly the best, in the set. A method that performs very well on most targets, but very poorly on a few, would still be recognized by this criteria. SELECTpro recovers the best model for 12 targets, selects a model with Δ*GDT*_*QA*1% _< 1% for 18 targets, and selects a model with Δ*GDT*_*QA*1% _< 10% for 66 targets. Group 091_1 (Ma-OPUS) also performs well, with 11, 18, and 61 targets in the respective categories. Only the 60 targets with Δ*GDT*_*QA*1% _< 10% of predictor 038_1 (GeneSilico) on its 85 target subset are better than SELECTpro in common subset comparison (58 for SELECTpro).

### The BLUNDER Measure Recovery of M_max_

How well does a QA predictor recover M_max_? The traditional metric to assess M_max _recovery is the *rank *of M_max_, and the average *rank *over many targets ($\bar{rank}$). While *rank *captures some important information, it ignores the redundancy of models and the quality of models ranked better than M_max_. Consider the following hypothetical situation: group *A *ranks M_max _10^th ^and all nine models ranked above it are redundant with Δ*GDT *of ~2.0, group *B *ranks M_max _5^th ^and the four models ranked above it are diverse with a Δ*GDT *between 10.0 and 20.0. Which group has done a better job of recovering M_max_? In this example, the *rank *metric favors group *B*, although group *A *ranks only a single redundant model above M_max_. In addition, the models ranked better than M_max _by group *A *have only slightly lower GDT-TS than M_max_, while the models ranked better than M_max _by group *B *are significantly worse than M_max_. To address these weaknesses of the *rank *metric, we introduce the BLUNDER metric, which focuses on the worst model ranked better than M_max _(the most embarrassing blunder). This measure is not affected by model redundancy and measures the quality of models ranked above M_max_. The BLUNDER metric is defined using the following notation, and used in the assessment of the recovery of M_max _and the corresponding Table 2 and Figure 1:

• M_*BLUNDER*_: The model with the minimum GDT-TS among models ranked better than M_max_.

• Δ*GDT*_*BLUNDER *_= GDT-TS(M_max_) - GDT-TS(M_*BLUNDER*_) : The GDT-TS difference between M_max _and M_*BLUNDER *_measures the size of the worst blunder.

• $\bar{\Delta GDT_{BLUNDER}}$ = ΣΔ*GDT*_*BLUNDER*_/*N*_*D *_: The average Δ*GDT*_*BLUNDER *_measures how well a method robustly recovers M_max _over many targets.

• Δ*GDT*_*BLUNDER*% _= Δ*GDT*_*BLUNDER*_/GDT-TS(M_max_) : The Δ*GDT*_*BLUNDER *_percentage allows for comparison across targets with different numbers of domains and difficulty levels.

Figure 1 contains graphs of the frequency of recovering M_max _using the *rank *(**A**) and Δ*GDT*_*BLUNDER*% _(**B**) measures on *SetComplete*. SELECTpro ranks M_max _first for 15 targets, in the top five for 39 targets, and in the top ten for 53 targets. SELECTpro's Δ*GDT*_*BLUNDER*% _values are less than 10% of GDT-TS(M_max_) for 40 targets and less than 20% for 63 targets. These results are best among all QA participants. The average M_max _recovery results are summarized in Table 2. The results columns are (1) average *rank *($\bar{rank}$) and (2) average Δ*GDT*_*BLUNDER *_($\bar{\Delta GDT_{BLUNDER}}$) on *SetAll *and *SetComplete*. The common subset results section also includes a column for the p-value of a paired t-test using Δ*GDT*_*BLUNDER *_(*p-value*). Rows are sorted separately for each dataset by *N*_*T *_first and then $\bar{\Delta GDT_{BLUNDER}}$. On *SetComplete *SELECTpro has a $\bar{\Delta GDT_{BLUNDER}}$ of 10.4. In common subset comparisons one group has a lower $\bar{rank}$: group 091_1 (Ma-OPUS) with $\bar{rank}$ of 16.8 on 94 targets compared to 17.4 for SELECTpro. On *SetAll *SELECTpro did not submit a score for M_max _of target T0356 (HHpred2_TS1) due to a processing error. In order to make complete common subset comparisons when possible we added in the SELECTpro score for HHpred2_TS1. SELECTpro ranks it 86^th ^and Δ*GDT*_*BLUNDER *_= 50.0. Both results are significantly worse than the SELECTpro averages.

### Pearson Correlation for Individual Proteins

The assessor evaluation of the quality assessment category [18] focused on the Pearson Correlation between the QA scores and GDT-TS. Here we use the Pearson Correlation only to highlight some of the difficulties for structure-based methods in dealing with incomplete models, as well as basic non-protein like structural features. Approximately half of the models in *SetAll *are incomplete, with backbone coordinates missing for one or more residues.

Incomplete models present a challenge to SELECTpro and other structure-based methods because the scores for each model are only comparable when calculated on coordinates for the same set of residues. Another issue is that some complete models have severe chain-breaks, severe steric clashes, or significant portions modeled only as extended chains. These local problems can overwhelm the energy of what may otherwise be a good model. Consensus methods do not suffer from these local structure problems. Given this rationale, one would expect structure-based methods to see the most improvement in terms of average Pearson Correlation on *SetComplete *relative to *SetAll*. Table 3 shows the average Pearson Correlation of five selected groups. Predictors 713_1 (Circle-QA), 633_1 (ProQ), and SELECTpro are structure-based MQAPs, while 634_1 (Pcons) is a consensus method and 556_1 (LEE) scored structures based on the GDT-TS similarity to their human Model 1 CASP7 prediction [18]. As expected, the structure-based MQAPs improve more than the structural similarity-based methods. The even greater increase in Pearson Correlation for SELECTpro can be accounted for by the failure to generate appropriate complete models for some of the incomplete models resulting in QA scores calculated on extended chains.

### Reranking Top Server Group Models

Predictors in CASP may submit up to five models, but CASP evaluation focuses on the model designated as Model 1. Clearly, the selection of Model 1 is critical in the CASP setting and for protein structure prediction in general. Figure 2 contains the results when SELECTpro is used to rerank the five models submitted by each of the top ten servers from CASP7, compared to each server's results. In the following assessment M_max-g _is the model with the highest GDT-TS of the five models submitted by a server. Figure 2 (**A**) shows that SELECTpro recovers M_max-g _more frequently than 8 of the top 10 server groups; in addition, when SELECTpro is used to select Model 1 the average GDT-TS increases for 7 of 10 sever groups; however, the increase is only statistically significant for 3 groups. SELECTpro improves using both criteria for the top 3 server groups (Zhang-Server, Pmodeller6, and ROBETTA). These results highlight the utility of SELECTpro for the task of model selection. The comparisons made here are fair because structure-based methods can be applied in the server setting to any number of models.

### Large Decoy Set Model Selection

Here we analyze SELECTpro's model selection capability on the large decoy sets for 16 small proteins from a recent I-TASSER benchmark set [19]. The I-TASSER prediction method generates 12500 to 20000 different backbone conformations. The complete decoy sets can be downloaded from [20]. The consensus method SPICKER [21] is used to cluster the models and a centroid model is built from the first cluster. A second round of simulation resolves the steric clashes in the centroid model and results in the final predicted model. The centroid model and final model are not part of the decoy set. In order to make a fair model selection comparison the decoy model closest to the centroid is used as I-TASSER's M_*QA*1_.

On the benchmark set SELECTpro has an average GDT-TS of 63.7, while I-TASSER has an average GDT-TS of 62.1. SELECTpro's average Δ*GDT*_*QA*1 _is 9.2 and I-TASSER's Δ*GDT*_*QA*1 _is 10.7. Figure 3 displays the GDT-TS results for the individual proteins in the benchmark set. Different symbols are used to indicate the GDT-TS of M_max _(□), the GDT-TS of SELECTpro's M_*QA*1 _(×), and the GDT-TS of I-TASSER's M_*QA*1 _(+) for each protein. A paired t-test of the hypothesis that SELECTpro and I-TASSER's mean performance are equal produces a p-value of .19, which is not statistically significant, but does give some evidence that SELECTpro can select a very good model from a large set of decoys at least well as an established method that utilizes consensus methods.

## Conclusion

A MQAP that can select the most native-like model from a set of possibilities has a variety of applications in protein structure prediction. The new quality assessment category introduced in CASP7 allows for the unbiased assessment of MQAPs on the models produced by automated predictors. This category allows researchers to focus on the model scoring aspect of protein structure prediction.

The results presented in this work demonstrate that SELECTpro, a structure-based model selection method, consistently selects one of the best models from the large diverse sets of models produced by automated predictors, across all levels of target difficulty. On these large diverse sets of models, SELECTpro also recovers the single most native-like model well compared to other methods. On the small sets of five models submitted for each target by the top automated predictors, in most cases SELECTpro selects better models than the predictors themselves.

Since SELECTpro and other structure-based methods score models independently, they can be incorporated into the model selection pipelines of individual protein structure prediction servers. For this reason, it may help predictors if the CASP organizers distinguished methods that score models independently from those that do not.

Consensus and structure-based methods can be combined to achieve improved results. For example, the meta-server method Pmodeller [22] combines consensus (Pcons [23]) and structure-based methods (ProQ [24]) to predict protein structures more accurately than either method in isolation. The assessment of the QA category by CASP assessors recognized the consensus method Pcons (group 634_1) for the high Pearson Correlation between their scores and model GDT-TS on most targets [18]. In their own assessment the authors of Pcons recognized that while consensus methods perform well in most cases, "when most of the models are incorrect and the few correct models are outliers a consensus based approach cannot be expected to make an optimal choice." [1] For instance, they identified three particular targets in CASP7 where their consensus method failed: T0283, T0350, and T0351 [1]. The Pcons average Δ*GDT*_*QA*1 _on these three targets is 30.8. The same research group's structure-based method ProQ (group 633_1) has an average Δ*GDT*_*QA*1 _of 17.2. In contrast, on these three targets SELECTpro has an average Δ*GDT*_*QA*1 _of only 7.1. This example highlights the potential of combining SELECTpro with existing model selection methods.

SELECTpro has been made publicly available as a server, where users may submit from 2 to 100 models for evaluation. In addition to the global confidence scores, the scores of individual energy terms are also returned to the user by email for each model submitted. SELECTpro is one of several protein structure tools in the SCRATCH suite of predictors [25], and is available through: .

## Methods

### Datasets

All of the comparative analysis in this work is performed on the server models and quality assessment predictions submitted in the CASP7 [26] experiment. The CASP QA experiment is particularly relevant for the evaluation of model selection methods for several reasons: (1) the QA predictors were blind to the true structures at the time of prediction making it impossible for methods to be tuned to improve results; (2) the set of proteins is diverse: the 95 targets range in size from 68 to 530 amino acids, come from a variety of organisms, and span the full range of prediction difficulty; (3) each target has more than 200 predicted models that contain the types of errors that occur in automated structure prediction; (4) the protein set is not selected by any of the participating QA groups; (5) the models are scored by a variety of methods and the results are publicly available. We perform analysis on the set of all models (*SetAll*) and a subset of models (*SetComplete*) that are complete and free of gross structural irregularities, as described below. All of the ABIpro models and some of the 3Dpro models were optimized using the exact energy function of SELECTpro. These models are removed because of the obvious bias towards these models. In recent CASP experiments the GDT-TS [27] has been used as the primary automatic structural similarity measure. The published GDT-TS values from the CASP7 website are the only structural similarity measure used in this work.

#### SetAll

**Figure 4:**
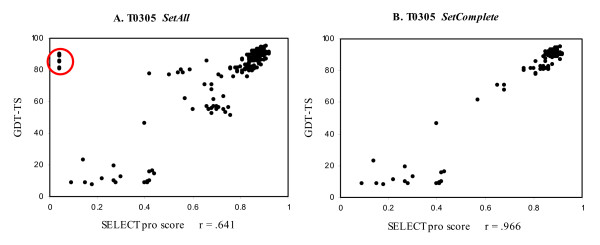
***SetAll *versus SetComplete**. Plots of SELECTpro scores versus GDT-TS scores for T0305 models from *SetAll *(**A**) and *SetComplete *(**B**). The Pearson correlation is .641 for *SetAll *and .996 for *SetComplete*. This large difference is mainly due to the extended chain models (circled in plot A) scored by SELECTpro due to an error in our use of Modeller to generate complete models from incomplete ones.

The *SetAll *dataset consists of the server models with a GDT-TS value published on the CASP7 website, a total of 23,423 models. To calculate a score on a protein model SELECTpro requires the backbone coordinates (N, C_α_, C) for all model residues as input. A total of 8,812 models in *SetAll *have only a C_α _trace or have no coordinates for one or more residues. Modeller8v1 [28-30] was used to generate complete models from the incomplete ones, and then the complete models were scored by SELECTpro. In most cases the complete models were built appropriately from the incomplete models; however, in some cases the final model was a fully extended chain due to an error in our application of Modeller. We failed to identify this problem until after the completion of the CASP7 competition. The SELECTpro scores versus GDT-TS scores for all models of target T0305 are displayed in plot A of Figure 4. The circled outliers with very low confidence scores and high GDT-TS scores are models that were incomplete and the complete models generated by Modeller were fully extended chains. The Pearson correlation on the set of all models for T0305 is .641. The SELECTpro scores versus GDT-TS scores for complete models only are displayed in plot B of Figure 4, and the Pearson correlation is .966.

#### SetComplete

The scores produced by SELECTpro are comparable on complete models of the same sequence. There is no standard for the handling of incomplete models and we assume that participating groups took a variety of approaches. Using only complete models ensures that the MQAP scores are calculated from the same coordinates. Thus, the models retained in *SetComplete *are screened first for completeness. Models missing backbone coordinates for one or more residues are removed. This leaves 14,611 models.

Structure-based MQAPs are susceptible to local structural irregularities in models, and will tend to score such models poorly. This is why methods developed to select near-native models from sets of decoys remove such models from consideration [31]. We apply additional filters (described below) for C_α_-C_α _clashes, C_α_-C_α _chain breaks, and expanded termini to remove an additional 1,217 models leaving 13,494 more plausible models in *SetComplete*.

The C_α_-C_α _clash model filter enforces a squared difference penalty for C_α_-C_α _distances less than 3.6 Ǻ. The distance between the C_α _atoms of residue *i *and *j *is denoted by *r*_*i*,Cα,*j*,Cα _and *N *is the protein length. The constant 13.52 in the threshold below corresponds to two severe clashes where *r*_*i*,Cα,*j*,Cα _= 1.0 Ǻ. Models with a sum of squared differences greater than 13.52 per 100 residues are filtered out.

$$\sum_{i>j}\max{\left\{0,3.6-r_{i,C_{\alpha },j,C_{\alpha }}\right\}}^{2}>13.52(N/100)$$

The C_α_-C_α _chain break model filter enforces a squared difference penalty for *r*_*i*,Cα,*i*+1,Cα _distances greater than 4.0 Ǻ. The constant 16.0 in the threshold below corresponds to a single chain break where *r*_*i*,Cα,*i*+1,Cα _= 8.0 Ǻ. Models with a sum of squared differences greater than 16.0 per 100 residues are filtered out.

$$\sum_{i}\max{\left\{0,r_{i,C_{\alpha },i+1,C_{\alpha }}-4.0\right\}}^{2}>16.0(N/100)$$

The expanded termini filter removes models where a large portion of the structure is modeled as expanded chain with no non-local interactions. The screening procedure is: scan from the N-terminus until three consecutive residues have a contact number of at least 10, and repeat from the C-terminus. The contact number of a residue is defined here as the number of other C_β _atoms within 10 Ǻ of the residue's C_β _[3]. If the sum of low contact number termini residues is at least 20% of *N*, the model is filtered out.

### Model Representations

#### Reduced representation

In the reduced representation the heavy backbone atoms, carbonyl oxygen, amide hydrogen (N, C_α_, C, O, H), and C_β _are represented explicitly. For glycine residues a pseudo C_β _is calculated. The side-chain atoms are represented by a single united point (centroid) [32,33]. The centroid is calculated as the mean of the position of the heavy side-chain atoms. For glycine and alanine the centroid (*CT*) is set to the C_β _atom. Only the heavy backbone atoms (N, C_α_, C) are used as input to SELECTpro and the positions of additional atoms and centroids are calculated from these.

#### All heavy-atom representation

In the all heavy-atom representation the centroid is removed and the heavy side chain atoms are represented explicitly. The side-chains are initially placed onto the backbone of the reduced representation in their most likely conformation according to the SCWRL backbone-dependent rotamer library [34]. The side-chain placements are then optimized using the SELECTpro all-atom energy terms (described below) in conjunction with the rotamer library.

### Energy Functions Overview

*E*_*REDUCED *_is the combined energy calculated from the reduced representation. *E*_*REDUCED *_is a linear combination of predicted (*E*_*PRED*-*SS*_, *E*_*PRED*-*SA*_, *E*_*PRED*-*CM*_), physical (*E*_*VDW*-*REP*_), and statistical (*E*_*CT*-*REP*_, *E*_*STAT*-*ENV*_*, E*_*STAT*-*PW*-*CI*_, *E*_*STAT*-*PW*-*CD*_, *E*_*ROG*_) terms:

*E*_*REDUCED *_= *w*_1_*E*_*PRED*-*SS *_+ *w*_2_*E*_*PRED*-*SA *_+ *w*_3_*E*_*PRED*-*CM *_+ *w*_4_*E*_*BETA *_+ *w*_5_*E*_*VDW*-*REP *_+ *w*_6_*E*_*CT*-*REP *_+ *w*_7_*E*_*STAT*-*ENV *_+ *w*_8_*E*_*STAT*-*PW*-*CI *_+ *w*_9_*E*_*STAT*-*PW*-*CD *_+ *w*_10_*E*_*ROG*_

*E*_*ALL*-*ATOM *_consists of the energy terms that depend on the all heavy-atom representation. *E*_*ALL*-*ATOM *_is a linear combination of the following physical terms:

*E*_*ALL*-*ATOM *_= *w*_11_*E*_*SC*-*HB *_+ *w*_12_*E*_*LEN*-*JONES *_+ *w*_13_*E*_*SOLVATION *_+ *w*_14_*E*_*ELECTRO*_

*E*_*FINAL *_is the sum of *E*_*REDUCED *_and *E*_*ALL*-*ATOM*_, and is used for the final scoring of models by SELECTpro. The individual energy terms are outlined briefly below and the detailed description of the novel terms follow in the remainder of this section. Underlined terms are adapted from previously described energy terms their details are included in the Appendix.

#### Parameter Weights

The parameter weights were determined by repeatedly varying individual weights and maximizing the sum of the GDT-TS of the lowest *E*_*FINAL *_models on a training set built from CASP6 protein domains. For each CASP6 protein domain a set of 500 decoy models was generated using fragment assembly with the RMSD to native as the dominant term in the objective function [3].

#### E_REDUCED_

*E*_*PRED*-*SS*_: predicted secondary structure

*E*_*PRED*-*ACC*_: predicted solvent accessibility

*E*_*PRED*-*CM*_: predicted contact map

*E*_*BETA*_: sheet formation

*E*_*BB*-*REP*_: backbone repulsion

*E*_*CT*-*REP*_: centroid repulsion

*E*_*STAT*-*ENV*_: residue environment potential [3]

*E*_*STAT*-*PW*-*CI*_: context independent pair-wise potential [3,16]

*E*_*STAT*-*PW*-*CD*_: context dependent pair-wise potential [6]

*E*_*ROG*_: compactness

#### E_ALL-ATOM_

*E*_*SC*-*HB*_: side-chain hydrogen bonding

*E*_*LEN*-*JONES*_: van der Waals forces [10]

*E*_*SOLVATION*_: solvation effects [35]

*E*_*ELECTRO*_: electrostatic interactions

Throughout this work the convention of all capital letters referring to global energy and all lower case referring to local energy is used. For instance, *E*_*PRED*-*CM *_refers to the global contact map energy and *E*_*pred*-*cm*_(*i,j*) refers to the contact map energy between residues *i *and *j*.

### Parameter notation used in energy equations

#### Model variables

*r*_*i*,*x*,*j*,*y*_: distance between atom *x *of residue *i *and atom *y *of residue *j*

*r*_*x*,*y*_: distance between atom *x *and atom *y*

v_*i*,*x*,*j*,*y*_: vector from atom *x *of residue *i *to atom *y *of residue *j*

u_*i*,*x*,*j*,*y*_: unit vector calculated from v_*i*,*x*,*j*,*y*_

*N*_*i*_: number of residues in contact with residue *i*, with contact defined as *r*_*i,Cβ,j,Cβ *_< 10 Ǻ

*phi*_*i*_: Phi angle of residue *i*

*psi*_*i*_: Psi angle of residue *i*

#### Protein specific input parameters

*aa*_*i*_: amino acid type of residue *i*

*ss*_*i*_: predicted secondary structure of residue *i *(H,E,C)

*acc*_*i*_: predicted solvent accessibility of residue *i *('e': exposed, '-', buried)

*cmap*_*i*,*j*_: predicted contact/non-contact between residues *i *and *j*, with contact defined as *r*_*i*,Cα,*j*,Cα _< 12 Ǻ

#### Protein independent parameters

*I*_*value*_: ideal parameter value for a given calculation

*σ*_*value*_: standard deviation value for a given calculation

*vdw*_*x*_: van der Waals radius of atom *x*

*vdw*_*x*+*y*_: *vdw*_*x *_+ *vdw*_*y*_

Ω_*stat*-*env*_: pre-calculated statistics for use in *E*_*STAT*-*ENV*_

Ω_*stat*-*pw*-*oi*_: pre-calculated statistics for use in *E*_*STAT*-*PW*-*CI*_

Ω_*stat*-*pw*-*od*_: pre-calculated statistics for use in *E*_*STAT*-*PW*-*CD*_

*D*_*min*,*pw*-*od*_: minimum interaction distance for centroid pairs used in *E*_*STAT*-*PW*-*CD*_

*D*_*max*,*pw*-*od*_: maximum interaction distance for centroid pairs used in *E*_*STAT*-*PW*-*CD*_

*D*_*min*-*CT*_: minimum distances between centroids of amino acid pairs observed in pdb_select25 [36].

### Reduced Representation Energy Term Details

The details of how the novel reduced representation energy terms are calculated are presented in this section. The predicted structural terms *E*_*PRED*-*SS*_, *E*_*PRED*-*ACC*_, and *E*_*PRED*-*CM *_and the β-strand pairing term, *E*_*BETA*_, are novel and unique to SELECTpro. Additional reduced representation terms are adapted from previously published work and their details are included in the Appendix.

#### Predicted structural features overview

The predicted structural feature predictions used in *E*_*PRED*-*SS*_, *E*_*PRED*-*ACC*_, and *E*_*PRED*-*CM *_come from the SCRATCH suite of predictors [25]. Each predictor is trained in a supervised fashion using curated non-redundant datasets extracted from the PDB [37]. The secondary structure (SSpro [38]) and solvent accessibility (ACCpro [39]) predictors use ensembles of 1D-RNN (one dimensional-recursive neural network) architectures [40]. The contact map predictor (CMAPpro [41]) uses ensembles of 2D-RNN architectures [40].

#### E_PRED-SS_: predicted secondary structure

The predicted secondary structure term *E*_*PRED*-*SS *_penalizes deviation of the torsion angles from the torsion angle parameters for helices and strands predicted by SSpro. There is no penalty for predicted coils. The parameter values for helix residues are: *I*_*Hφ *_= -65.3, σ_*Hφ *_= 11.9, *I*_*Hψ *_= -39.4, σ_*Hψ *_= 11.3. The parameter values for strand residues are: *I*_*Eφ *_= -135.0, σ_*Eφ *_= 15.0, *I*_*Eψ *_= 135.0, σ_*Eψ *_= 15.0. Only torsion angles that are more than two standard deviations from the ideal are penalized, with the penalty defined as follows:

$$\begin{array}{l} E_{PRED-SS}=\sum_{ss_{i}=H}E_{pred-helix}(i)+\sum_{ss_{j}=E}E_{pred-strand}(j)\\ E_{pred-helix}(i)=\sqrt{\Theta_{H\Phi }(i){\left(\left|phi_{i}-I_{H\Phi }\right|-2\sigma_{H\Phi }\right)}^{2}+\Theta_{H\Psi }(i){\left(\left|psi_{i}-I_{H\Psi }\right|-2\sigma_{H\Psi }\right)}^{2}}\\ \Theta_{H\Phi }(i)=\left\{ \begin{array}{l} 1\text{, if}\left|phi_{i}-I_{H\Phi }\right|>2\sigma_{H\Phi }\\ \text{0, otherwise} \end{array}\right.\\ \Theta_{H\Psi }(i)=\left\{ \begin{array}{l} 1\text{, if}\left|psi_{i}-I_{H\Psi }\right|>2\sigma_{H\Psi }\\ \text{0, otherwise} \end{array}\right. \end{array}$$

The definition of *E*_*pred*-*strand*_(*j*) is equivalent to *E*_*pred*-*helix*_(*i*), but with *I*_*Eφ*_, σ_*Eφ*_, *I*_*E*ψ _and σ_*E*ψ _in place of the corresponding helical values.

#### E_PRED-ACC_: predicted solvent accessibility

The solvent accessibility predictor ACCpro predicts the percent of solvent accessibility in 5% increments for each residue. Using 25% exposure as a binary threshold the accuracy of the predictor is ~77% [39]. The binary exposure ('*e*')/burial ('-') prediction is used as the predicted solvent accessibility for *E*_*PRED*-*ACC*_. In the reduced representation the solvent accessibility of residue *i *is estimated by its contact number (*N*_*i*_), where *N*_*i *_> 16 is considered buried [3]. If the predicted status of a residue is not realized in the model, the penalty is calculated as:

$$\begin{array}{l} E_{PRED-ACC}=\sum_{i}E_{pred-acc}(i)\\ E_{pred-acc}(i)=\{\begin{array}{ll} {\left(17-N_{i}\right)}^{2}\text{, } & \text{if }acc_{i}='-'\text{and }N_{i}\leq 16\\ {\left(N_{i}-16\right)}^{2}\text{,} & \text{if }acc_{i}='e'\text{and }N_{i}>16\\ 0, & \text{otherwise} \end{array} \end{array}$$

#### E_PRED-CM_: predicted contact map

The contact map predictor CMAPpro predicts the probability of contact or non-contact between C_α _atoms, with a contact threshold of 12 Å. The strategy utilized to infer predicted contacts from the probability matrix [41] results in maps that are sparse when compared to those of real proteins; thus, unrealized contacts are penalized while non-contacts are not. The constant 1.0 is added to the penalty to ensure that all unrealized contacts make a significant contribution to *E*_*PRED*-*CM*_.

$$\begin{array}{l} E_{PRED-CM}=\sum_{j>i}\Theta \;(i,j)E_{pred-cm}(i,j)\\ \Theta \;(i,j)=\{\begin{array}{ll} 1, & \text{if }r_{i,C_{\alpha },j,C_{\alpha }}>I_{cm-thresh}\\ 0, & \text{otherwise} \end{array}\\ E_{pred-cm}(i,j)=cmap_{i,j}\left(1.0+\frac{r_{i,C_{\alpha },j,C_{\alpha }}^{2}}{I_{cm-thresh}^{2}}\right) \end{array}$$

**Figure 5:**
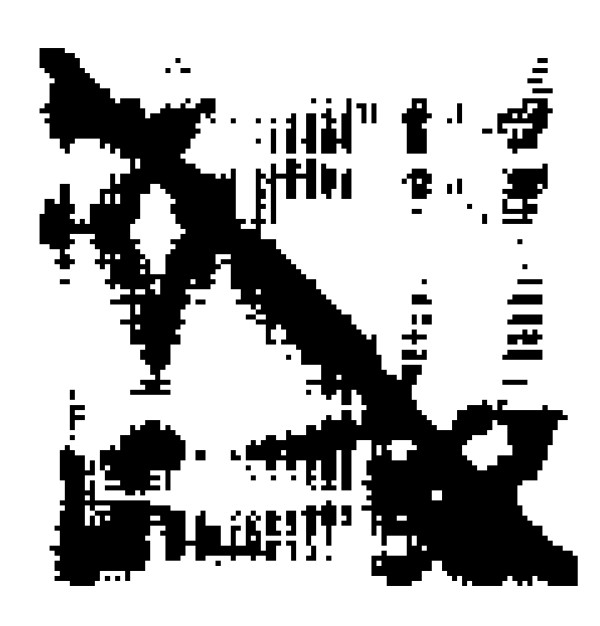
**Contact map comparison**. True contact map of target T0304 in lower left, predicted contacts upper right. Contact is defined as Cα atoms within 12 Å. For predicted contacts with a sequence separation of at least six, 651 of 915 (71%) are correct.

The predicted contact map can help identify the highest GDT-TS models in the set, even when they are not highly similar to native. A good example of this is CASP7 target T0304 is a 122 residue α/β protein where the highest GDT-TS model in the set is Zhang-Server_TS1 (GDT-TS = 45.55). Most secondary structure predictors (including SSpro) failed to predict the first two strands making this target especially difficult. No QA method ranked the highest GDT-TS model first; however, SELECTpro ranked it second and the model ranked first by SELECTpro (T0304.Zhang-Server_TS4) has the second highest GDT-TS. These models have the lowest *E*_*PRED*-*CM *_of any models in the set, but the native structure has an even lower *E*_*PRED*-*CM*_. Figure 5 compares the native and predicted contact maps for target T0304.

#### E_BETA_: strand pairing

The formation of hydrogen bonds between the residues of *β*-strand partners is a major determinant of the tertiary structure of *β *and *α/β *proteins. The *β *hydrogen bonding treatment described here favors realistic strand pairing and sheet formation. The treatment also efficiently accommodates bulges in strands because it does not force the register between two paired strands. *E*_*BETA *_is the global strand pairing energy that penalizes the hydrogen bonding of *β *residues between strand pairs. *E*_*beta*-*sp*_(*β*_*k*_→*β*_*w*_) is the strand pairing energy of strand *β*_*k *_to strand *β*_*w*_. *E*_*beta*-*sp *_is only commutative if the two strands have the same length. *E*_*beta*-*hb*_(*i,j*) is the hydrogen bonding penalty between residues *i *and *j*.

*E*_*beta*-*sp *_is calculated for all possible strand pairings, but only the two lowest energies from each strand are used in *E*_*BETA*_. Other strand-strand interactions are ignored. In the equations below *S *is the set of all strands in the protein, *β*_*m*1 _is the strand with the minimum pairing energy from *β*_*k*_, and *β*_*m*2 _is the strand with the next lowest pairing energy from *β*_*k*_. If the strand count is less than six at least two of the strands must be edge strands. This is accounted for by only considering the single best strand partner for two strands.

$$\begin{array}{l} E_{BETA}=\sum_{\beta_{k}\in S}E_{beta-sp}(\beta_{k}\to \beta_{m1})+E_{beta-sp}(\beta_{k}\to \beta_{m2})\\ \beta_{m1}=\{\beta_{x}:\underset{\beta_{x}\in S/\{\beta_{k}\}}{\min}E_{beta-sp}(\beta_{k}\to \beta_{x})\}\\ \beta_{m2}=\{\beta_{y}:\underset{\beta_{y}\in S/\{\beta_{k},\beta_{m1}\}}{\min}E_{beta-sp}(\beta_{k}\to \beta_{y})\} \end{array}$$

In the equations for *E*_*beta*-*sp *_below, *S*_*k *_is the set of all residues in strand *β*_*k*_. Each time *E*_*beta*-*hb *_is calculated the pair (*i,j*) is chosen with *i *from *S*_*k *_and *j *from *S*_*w*_, such that *E*_*beta*-*hb *_is minimized. Then residue *i *is removed from *S*_*k*_, and residue *j *is removed from *S*_*w*_. *E*_*beta*-*hb *_is calculated once for each residue in *S*_*k*_. If *S*_*k *_has more residues than *S*_*w *_each unpaired residue is given maximum penalty of *E*_*beta*-*hb*_.

$$\begin{array}{l} E_{beta-sp}(\beta_{k}\to \beta_{w})=\sum_{}^{S_{k}\neq \emptyset ,}E_{beta-hb}(i,j)\\ \left(i,j)=\{(x,y):\underset{x\in S_{k},y\in S_{w}}{\min}E_{beta-hb}(x,y)\right\}\\ S_{k}=S_{k}/\{i\},\;S_{w}=S_{w}/\{j\} \end{array}$$

Between two anti-parallel strand partners, only every other pair of residues is hydrogen bonded. For the pairs that are not hydrogen bonded, a pseudo-bonding calculation is used. The hydrogen bonding energy and pseudo-bonding energy are both calculated and the minimum of the two is used in *E*_*beta*-*hb*_(*i,j*).

If residues *i *and *j *are paired in parallel strands, either *i *forms hydrogen bonds with *j*-1 and *j*+1, or *j *forms hydrogen bonds with *i*-1 and *i*+1. No hydrogen bonds are formed between the atoms of residues *i *and *j*. The hydrogen bonding energy is calculated for both possible conformations and only the minimum of the two is used in *E*_*beta*-*hb*_(*i,j*).

$$E_{beta-hb}(i,j)=\{\begin{array}{ll} \text{min\{}\Phi \text{(}i\to j)+\Phi \text{(}j\to i),\Phi_{ps}\text{(}i\to j)+\Phi_{ps}\text{(}j\to i)\}, & \text{if strands are anti-parallel}\\ \text{min\{}\Phi \text{(}i\to j+1)+\Phi \text{(}j-1\to i),\Phi \text{(}i-1\to j)+\Phi \text{(}j\to i+1)\}, & \text{if strands are parallel} \end{array}$$

Φ(*a*→*d*) is the directional energy calculation for a single hydrogen bond where *a *is the index of the acceptor residue and *d *is the index of the donor residue. Three geometrical measures are used to estimate the strength of hydrogen bonds: the distance between the acceptor and the hydrogen atoms (*r*_*a*,*O*,*d*,*H*_), the angle at the acceptor atom (u_*a*,*C*,*a*,*O *_· u_*a*,*O*,*d*,*H*_), and the angle between the acceptor and donor atom vectors (u_*a*,*C*,*a*,*O *_· u_*d*,*N*,*d*,*H*_). The distance and acceptor atom angle parameters are motivated by the orientation-dependent hydrogen bonding potential described in [42]. The following parameters were set based on idealized hydrogen bonding between β residues, with standard deviation values set such that two standard deviations approximate the cut-off in true hydrogen bonds. The ideal distance from hydrogen atom to accepting oxygen is *I*_*hb*-*dist *_= 1.9 Ǻ, with standard deviation σ_*hb*-*dist *_= 0.5 Ǻ. The ideal angle at the acceptor atom is 0°, so the ideal (u_*a*,*C*,*a*,*O *_· u_*a*,*O*,*d*,*H*_) is *I*_*acc*-*dp *_= 1.0, with standard deviation σ_*acc*-*dp *_= 0.11. The ideal angle between the acceptor and donor atom vectors is 180°, so the ideal (u_*a*,*C*,*a*,*O *_· u_*d*,*N*,*d*,*H*_) is *I*_*acc*-*don*-*dp *_= -1.0, with standard deviation σ_*acc*-*dp *_= 0.15. The parameters for pseudo-bonded residues are as follows: the ideal distance for *r*_*a*,*O*,*d*,*H *_is *I*_*ps*-*hb*-*dist *_= 7.9 Ǻ, *I*_*ps*-*acc*-*dp *_= -1.0, and *I*_*ps*-*acc*-*don*-*dp *_= -1.0. The standard deviations from the corresponding hydrogen bonding parameters above are used in Φ_ps_(*a*→*d*).

$$\begin{array}{l} \Phi (a\to d)=\left\{ \begin{array}{l} \begin{array}{cc} 54.0, & \text{if }r_{a,O,d,H}>7.0 \end{array}\\ \Psi (r_{a,O,d,H},I_{hb-dist},\sigma_{hb-dist})\\ +\Psi {\text{(u}}_{a,C,a,O}•{\text{u}}_{d,N,d,H},I_{acc-don-dp},\sigma_{acc-don-dp})\\ \begin{array}{cc} +\Psi {\text{(u}}_{a,C,a,O}•{\text{u}}_{a,O,d,H},I_{acc-dp},\sigma_{acc-dp}), & \text{otherwise} \end{array} \end{array}\right.\\ \Phi_{ps}(a\to d)=\left\{ \begin{array}{l} \begin{array}{cc} 54.0, & \text{if }r_{a,O,d,H}>10.0 \end{array}\\ \Psi (r_{a,O,d,H},I_{ps-hb-dist},\sigma_{ps-hb-dist})\\ +\Psi {\text{(u}}_{a,C,a,O}•{\text{u}}_{d,N,d,H},I_{acc-don-dp},\sigma_{acc-don-dp})\\ \begin{array}{cc} +\Psi {\text{(u}}_{a,C,a,O}•{\text{u}}_{a,O,d,H},I_{ps-acc-dp},\sigma_{acc-dp}), & \text{otherwise} \end{array} \end{array}\right. \end{array}$$

The penalty for the observed value (*x*) increases up to 6 standard deviations from the ideal value (*μ*).

$$\Psi (x,\mu ,\sigma )=\{\begin{array}{ll} \frac{{\left(x-\mu \right)}^{2}}{2\sigma^{2}}, & \text{if }\left|x-\mu \right|<6\sigma \\ \frac{{\left(6\sigma \right)}^{2}}{2\sigma^{2}}=18.0, & \text{otherwise} \end{array}$$

### All-Atom Energy Term Details

The all-atom energy terms depend on atom-atom interactions when all heavy atoms are included in the model. In the all-atoms energy equations *x *and *y *refer to atoms in the model and the residue positions are not referenced. The van der Waals radii and well-depths (*ε*_*x*_, used in *E*_*LEN*-*JONES*_) come from the CHARMM19 parameter set [43]. The side-chain hydrogen bonding term, *E*_*SC*-*HB*_, is described in detail here because it is unique to SELECTpro. The details of *E*_*LEN*-*JONES*_, *E*_*SOLVATION*_, and *E*_*ELECTRO *_are provided in the Appendix.

#### E_SC-HB_: side-chain hydrogen bonding

*E*_*SC*-*HB *_penalizes unsatisfied hydrogen bond donor and acceptor atoms that are at least partially buried. There is no penalty for fully exposed donor or acceptor atoms. Exposure percent ($\Delta G_{x}^{slv}$ %) is calculated as $\Delta G_{x}^{slv}/\Delta G_{x}^{ref}$. The definitions of $\Delta G_{x}^{slv}$ and $\Delta G_{x}^{ref}$ are provided in the description of *E*_*SOLVATION *_in the Appendix. Atoms at least 75% exposed are considered fully exposed and atoms less than 25% exposed are considered fully buried. For 25% <$\Delta G_{x}^{slv}$% < 75% the penalty weight is reduced linearly from 1.0 at 25% to 0 at 75%. The ideal distance from the acceptor atom to donor atom is *I*_*hb*-*da*-*dist *_= 2.9 Ǻ. In the equations below *donors *is the set of all side-chain hydrogen donor atoms and *acceptors *is the set of all side-chain hydrogen acceptor atoms.

$$\begin{array}{l} E_{SC-HB}=\sum_{x\in acceptors}E_{hb-acc}(x)+\sum_{x\in donors}E_{hb-don}(x)\\ E_{hb-acc}(x)=\lambda (x)\underset{y\in donors}{\min}{\left|r_{x,y}-I_{hb-da-dist}\right|}^{2}\\ E_{hb-don}(x)=\lambda (x)\underset{y\in acceptors}{\min}{\left|r_{x,y}-I_{hb-da-dist}\right|}^{2}\\ \lambda (x)=\{\begin{array}{ll} 1 & \text{if }\Delta G_{x}^{slv}\%<.25\\ 0 & \text{if }\Delta G_{x}^{slv}\%>.75\\ 2(.75-\Delta G_{x}^{slv}\%) & \text{otherwise} \end{array} \end{array}$$

## Appendix

In the interest of completeness and reproducibility we include the details of the energy terms that are adapted from previous work.

### Reduced Representation Energy Term Details

#### E_BB-REP_: backbone repulsion

This term penalizes steric clashes between non-bonded atoms explicitly represented in the reduced representation. The penalty for overlapping atoms is the overlap distance squared as defined here:

$$\begin{array}{l} E_{BB-REP}=\sum_{j>i}E_{bb-rep}(i,j)\\ E_{bb-rep}(i,j)=\sum_{x}\sum_{y}\Theta (i,x,j,y){\left(vdw_{x+y}-r_{i,x,j,y}\right)}^{2}\\ \Theta \;(i,x,j,y)=\{\begin{array}{ll} 1, & \text{if }r_{i,x,j,y}<vdw_{x+y}\\ 0, & \text{otherwise} \end{array} \end{array}$$

#### E_CT-REP_: centroid repulsion

A centroid-centroid repulsive term is used to reduce the overcrowding of side-chains in the reduced representation. The minimum distance between two centroids in the calculation is the minimum observed for each pair of residue types – *D*_*CT*-*min*_(*aa*_*i*_,*aa*_*j*_) – in pdb_select25. The penalty for centroid-centroid overlaps is defined as the overlap distance squared:

$$\begin{array}{l} E_{CT-REP}=\sum_{j>i}\Theta (i,j){\left[D_{CT-\min}(aa_{i},aa_{j})-r_{i,CT,j,CT}\right]}^{2}\\ \Theta (i,j)=\{\begin{array}{ll} 1, & \text{if }r_{i,CT,j,CT}<D_{CT-\min}(aa_{i},aa_{j})\\ 0, & \text{otherwise} \end{array} \end{array}$$

#### E_STAT-ENV_: residue environment potential

The motivation for this term is to model the hydrophobic effect. The level of burial for each residue in the model is estimated by the number of other C_β _atoms within 10 Ǻ (the contact number *N*_*i*_) [3]. The values in the table Ω_*stat*-*env *_reflect the likelihood of observing a particular *N*_*i *_for each residue type. For model residues near both termini the contact number is artificially increased to account for the missing neighbors along the chain.

$$\begin{array}{l} E_{STAT-ENV}=\sum_{i}\Omega_{stat-env}(aa_{i},N_{i}^{*})\\ N_{i}^{*}=\{\begin{array}{ll} N_{i}+4-i\text{,} & \text{if }i<4\\ N_{i}+4-\left|i-N\right|\text{,} & \text{if }\left|i-N\right|<4\\ N_{i}\text{,} & \text{otherwise} \end{array} \end{array}$$

#### E_STAT-PW-CI_: context independent pair-wise interactions

This context independent pair-wise potential comes from Equation 6 of [3]. The potential considers the likelihood of observing the pair of centroids in a given distance bin relative to the background, with distance bins of < 5, 5–7, 7–10, 10–12, and > 12 Å. The advantage of a context independent pair-wise potential is that it is less vulnerable to over-fitting by a conformational search because of its generality.

$$\begin{array}{l} E_{STAT-PW-CI}=\sum_{j>i}E_{stat-pw-ci}(i,j)\\ E_{stat-pw-ci}(i,j)=\Omega_{stat-pw-ci}[aa_{i},aa_{j},r_{bin}(i,j)]\\ r_{bin}(i,j)=\{\begin{array}{ll} 0-5 & \text{if }r_{i,CT,j,CT}\leq 5.0\\ 5-7 & \text{if}5.0<r_{i,CT,j,CT}\leq 7.0\\ 7-10 & \text{if }7.0<r_{i,CT,j,CT}\leq 10.0\\ 10-12 & \text{if }10.0<r_{i,CT,j,CT}\leq 12.0\\ 12+ & \text{if }r_{i,CT,jCT}>12.0 \end{array} \end{array}$$

#### E_STAT-PW-CD_: context dependent pair-wise potential

This context specific pair-wise potential is from [6]. This pair-wise potential depends on the local structure and relative orientation of both amino acids in the interaction. The statistics are calculated independently for each combination of local structures and relative orientations. At each position the local structure is considered either compact or open and the relative orientation is determined by the dot product of the C_α _to C_β _unit vectors of each residue and divided into three classes: parallel, anti-parallel, and intermediate.

$$\begin{array}{l} E_{STAT-PW-CD}=\sum_{j>i}E_{stat-pw-cd}(i,j)\\ E_{stat-pw-od}(i,j)=\Theta (i,j)\Omega_{stat-pw-od}[aa_{i},aa_{j},\lambda (i),\lambda (j),\Phi (i,j)]\\ \Theta (i,j)=\{\begin{array}{ll} 1, & \begin{array}{cc} \text{if }r_{i,CT,j,CT}>D_{\min,pw-od}[aa_{i},aa_{j},\lambda (i),\lambda (j),\Phi (i,j)] & \text{and} \end{array}\\ & r_{i,CT,j,CT}<D_{\max,pw-od}[aa_{i},aa_{j},\lambda (i),\lambda (j),\Phi (i,j)]\\ 0, & \text{otherwise} \end{array}\\ \lambda (i)=\{\begin{array}{ll} \text{compact}, & \text{if }r_{i-1,C_{\alpha },i+1,C_{\alpha }}<6.0\\ \text{open,} & \text{otherwise} \end{array}\\ \Phi (i,j)=\{\begin{array}{ll} \text{parallel}, & {\text{if u}}_{i,C_{\alpha },i,C_{\beta }}•{\text{u}}_{j,C_{\alpha },j,C_{\beta }}>.5\\ \text{antiparallel}, & {\text{if u}}_{i,C_{\alpha },i,C_{\beta }}•{\text{u}}_{j,C_{\alpha },j,C_{\beta }}<-.5\\ \text{intermediate}, & \text{otherwise} \end{array} \end{array}$$

#### E_ROG_: compactness

The radius of gyration is a simple measure of the global compactness of a domain. *E*_*ROG *_penalizes models that are less compact than expected according to [44]. If the radius of gyration of the model (λ) is less than the expected value (2.2*N*^.38^), there is no penalty. If it is greater, then the penalty is the squared difference between observed and expected. In the equation below *r*_*i*,*mean *_is the distance between the C_α _of residue *i *and the mean of all C_α_s in the model.

$$\begin{array}{l} E_{ROG}=\Theta (\lambda -2.2N^{.38}{)}^{2}\\ \lambda =\sqrt{\frac{\sum r_{i,mean}^{2}}{N}}\\ \Theta =\{\begin{array}{ll} 1, & \text{if }\lambda >2.2N^{.38}\\ 0, & \text{otherwise} \end{array} \end{array}$$

### All-Atom Energy Term Details

#### E_LEN-JONES_: van der Waals forces

A fundamental characteristic of native globular protein structures is their efficient steric packing of atoms in the protein core. A Lennard-Jones 12-6 potential with damped repulsion (*E*_*LEN*-*JONES*_) is used to measure the quality of steric packing. *E*_*LEN*-*JONES *_is the sum of local energy calculations *E*_*len*-*jones*_(*x,y*) performed on all pairs of non-bonded atoms. Since the repulsive portion of the standard Lennard-Jones 12-6 potential will overwhelm the entire energy function with a single significant atom-atom clash – repulsion is handled by a linear ramp from 0 to 10 as shown in the equation below [10]. Since *E*_*len*-*jones *_= 0 when (*vdw*_*x*,*y*_/*r*_*x*,*y*_) = $\sqrt[{6}]{2}$ independent of atom types, the switch to a linear ramp occurs when (*vdw*_*x*,*y*_/*r*_*x*,*y*_) > $\sqrt[{6}]{2}$.

$$\begin{array}{l} E_{LEN-JONES}=\sum_{y>x}E_{len-jones}(x,y)\\ E_{len-jones}(x,y)=\{\begin{array}{ll} 10.0\left(1-\frac{\sqrt[{6}]{2}}{vdw_{x,y}/r_{x,y}}\right)\text{,} & \text{if }\left(vdw_{x,y}/r_{x,y}\right)\;>\sqrt[{6}]{2}\\ \sqrt{\varepsilon_{x}\varepsilon_{y}}\left[{\left(\frac{vdw_{x,y}}{r_{x,y}}\right)}^{12}-2{\left(\frac{vdw_{x,y}}{r_{x,y}}\right)}^{6}\right], & \text{otherwise} \end{array} \end{array}$$

#### E_SOLVATION_: solvation effects

Solvation energy is calculated using the implicit solvation model described in [35] with the following adjustment: for overlapping atoms, the sum of their van der Waals radii is used in the calculation in place of the observed atom-atom distance in the model. This restricts the amount a single atom can contribute to the burial of another atom. Without this adjustment overlapping atoms will bias the calculation to indicate an atom is more buried than it would be otherwise. In the solvation model $\Delta G_{x}^{slv}$ is the observed solvation free energy of atom *x *in the model, calculated as the free energy of the fully exposed atom ($\Delta G_{x}^{ref}$) minus the reduction in solvation caused by the surrounding atoms. $\Delta G_{x}^{free}$ was determined empirically by setting it equal to $\Delta G_{x}^{ref}$ and increasing its magnitude until $\Delta G_{x}^{slv}$ of deeply buried atoms became zero. *λ*_*x *_is the correlation length of atom *x*. *V*_*y *_is the volume neighboring atom *y*. The values of these parameters come from [3535], with the exception of $\Delta G_{x}^{ref}$[45]. The equation for $\Delta G_{x}^{slv}$ below is the combination of Equations 5,6, and 7 of [35], with the atom overlap adjustment.

$$\begin{array}{l} E_{SOLVATION}=\sum_{x}\Delta G_{x}^{slv}\\ \Delta G_{x}^{slv}=\Delta G_{x}^{ref}-\frac{\Delta G_{x}^{free}}{2\lambda_{x}\pi \sqrt{\pi }}\sum_{x\neq y}\frac{e^{-\left[{\left(\frac{r_{x,y}^{*}-vdw_{x}}{\lambda_{x}}\right)}^{2}\right]}V_{y}}{r_{x,y}^{*2}}\\ r_{x,y}^{*}=\{\begin{array}{ll} vdw_{x+y}, & \text{if }r_{x,y}<vdw_{x+y}\\ r_{x,y}\text{,} & \text{otherwise} \end{array} \end{array}$$

#### E_ELECTRO_: electrostatics

Electrostatic interactions between charged atoms are treated by simple repulsion and attraction according to inverse distance squared. The use of distance squared rather than linear distance encourages the formation of salt bridges in the models. There is a correction for atom-atom distance below the minimum realistic value. The ideal distance between oppositely charged atoms is *I*_*hb*-*da*-*dist *_= 2.75 Ǻ. In the equations below *pos *is the set of all positively charged atoms and *neg *is the set of all negatively charged atoms.

$$\begin{array}{l} E_{ELECTRO}=\sum_{y>x\in pos\cup neg}\Theta (x)\Theta (y)/r_{x,y}^{*2}\\ \Theta (x)=\{\begin{array}{ll} 1, & \text{if }x\in pos\\ -,1 & \text{if }x\in neg \end{array}\\ r_{x,y}^{*}=\{\begin{array}{ll} I_{e\_dist}, & \text{if }r_{x,y}<I_{e\_dist}\\ r_{x,y}\text{,} & \text{otherwise} \end{array} \end{array}$$

## Availability and requirements

• **Project home page: **

• **Operating system: **linux for stand alone version, server is platform independent

• **Programming language: **C++ and Perl

• **Software requirements: **Perl

• **Disk space requirements: **1.6 Gb for full version, 13 Mb without feature predictors

## Authors' contributions

AR and PB designed the novel energy terms. AR implemented the methods and carried out the experiments. AR and PB authored the manuscript. Both authors approved the manuscript.
